# Near-Ultraviolet Indoor Black Light-Harvesting Perovskite Solar Cells

**DOI:** 10.1021/acsaem.2c01560

**Published:** 2022-11-17

**Authors:** Arivazhagan Valluvar Oli, Zinuo Li, Yu Chen, Aruna Ivaturi

**Affiliations:** † Smart Materials Research and Device Technology (SMaRDT) Group, Department of Pure and Applied Chemistry, University of Strathclyde, Thomas Graham Building, Glasgow G1 1XL, U.K.; ‡ Department of Physics, University of Strathclyde, Glasgow G4 0RE, U.K.

**Keywords:** perovskite solar cells, indoor light harvesting, near-UV light harvesting, interface engineering, IoT sensors

## Abstract

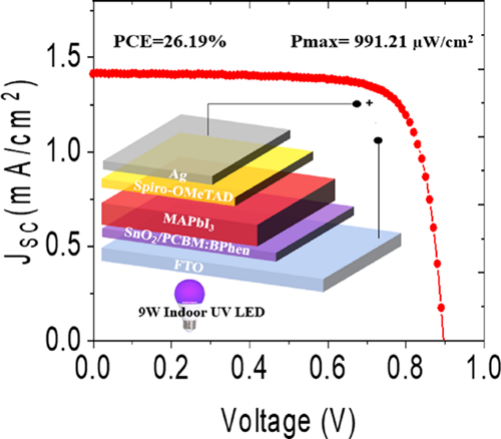

Indoor light-energy-harvesting solar cells have long-standing history with perovskite solar cells (PSCs) recently emerging as potential candidates with high power conversion efficiencies (PCEs). However, almost all of the reported studies on indoor light-harvesting solar cells utilize white light in the visible wavelength. Low wavelength near-ultraviolet (UV) lights used under indoor environments are not given attention despite their high photon energy. In this study, perovskite solar cells have been investigated for the first time for harvesting energy from a commercially available near-UV (UV-A) indoor LED light (395–400 nm). Also called black lights, these near-UV lights are commonly used for decoration (e.g., in bars, pubs, aquariums, parties, clubs, body art studios, neon lights, and Christmas and Halloween decorations). The optimized perovskite solar cells with the *n*–*i*–*p* architecture using the CH_3_NH_3_PbI_3_ absorber were fabricated and characterized under different illumination intensities of near-UV indoor LEDs. The champion devices delivered a PCE and power output of 20.63% and 775.86 μW/cm^2^, respectively, when measured under UV illumination of 3.76 mW/cm^2^. The devices retained 84.10% of their initial PCE when aged under near-UV light for 24 h. The effects of UV exposure on the device performance have been comprehensively characterized. Furthermore, UV-stable solar cells fabricated with a modified electron transport layer retained 95.53% of its initial PCE after 24 h UV exposure. The champion devices delivered enhanced PCE and power output of 26.19% and 991.21 μW/cm^2^, respectively, when measured under UV illumination of 3.76 mW/cm^2^. This work opens up a novel direction for energy harvesting from near-UV indoor light sources for applications in microwatt-powered electronics such as internet of things sensors.

## 1 Introduction

Solar cells have long standing history for harvesting energy from indoor artificial sources with recent renewed interest because of the developments of self-powered electronics.^1−4^ Besides extensive power applications of solar cells under outdoor conditions, indoor energy-harvesting solar cells are promising for self-powered microwatt energy-harvesting devices [such as sensors, actuators for internet of things (IoTs)], with potential to reduce the use and frequent replacements of batteries.^4−8^ Among the different types of solar cell materials, metal halide perovskites exhibit extraordinary optoelectronic properties such as tunable direct band gaps, high charge carrier mobility, and strong panchromatic absorption for both outdoor and indoor solar cell applications.^9−12^ Furthermore, perovskite solar cells (PSCs) offer cost-effective solution processing for large area fabrication toward commercialization.^13−15^ While the PSCs have achieved power conversion efficiencies (PCE) exceeding 25% under 1 sun condition,^16^ the devices are also showing a sky-rocketed PCE of over 36% under 1000 lux 15 W white LED,^17^ which makes them promising candidates for indoor applications requiring microwatt energy. It is worth noting that the charge carrier generation from indoor artificial light is relatively low as the intensity is less than 1 sun, which directly affect the short circuit current density of indoor light-harvesting solar cells.^18^ The enhanced PCE for indoor PSCs have been achieved through interface and compositional engineering which also prevents the devices from degradation against environmental stress.^9,19,20^ Interface engineering in general reduces the surface defects and improves the band alignment between the layers to facilitate enhanced charge carrier extraction. For example, postdeposition of phenethylammonium iodide salt on the wide band gap perovskite layer improved the absorption of the film for indoor application, and the resulting solar cells delivered a high open-circuit voltage of 0.9 V with a current density of 18 μW/cm^2^ under 200 lux (cool white LED).^8^ In a seminal work, the optimal band gap of perovskite solar cells were tailored to 1.9 eV by incorporating bromide (Br^–^) ions into the CH_3_NH_3_PbI_3_ perovskite absorber, which delivered a PCE >25% under white LED.^9^ Ionic liquid of 1-butyl-3-methylimidazolium tetrafluoroborate ([BMIM]BF_4_) employed as an interface modification layer in PSCs resulted in a PCE exceeding 35% under 1000 lux Fluorescent lamp.^20^ A path toward accelerating indoor perovskite photovoltaics market uptake for households, businesses, wearable, and industry applications has been recently reported.^21^ However, almost all of the studies on indoor light-harvesting solar cells reports only device performance under white light, mainly the visible wavelength region.^22−24^ In general, in addition to air and moisture, UV lights are reported to be detrimental for PSC stability, and various strategies such as composition and interface passivation strategies have been reported to improve the photostability either by blocking the UV radiation or improving the interface stability to prevent degradation.^25−29^ Interestingly, it has been reported that the performance of UV (365 nm, 7.6 mW/cm^2^) degraded PSCs can be recovered with >60% of initial PCE upon subsequent 1 sun light illumination (under an inert atmosphere) owing to the neutralization and resolving of accumulated trap states by free charge carriers generated by 1 sun light soaking.^30^ Charge-selective contacts and their interfaces with perovskites have been identified to play vital role in enhancing UV stability of PSCs. For instance, the use of ZnTiO_3_ electron transport layer in PSCs is reported to hold >90% of the initial PCE after 100 h of continuous UV (365 nm, 8 mW/cm^2^) exposure.^31^ A biaxially extended octithiophene-based conjugated polymer used as a hole transport layer in PSCs is shown to filter a large portion of UV radiation (365 nm, 5 mW/cm^2^) and prevent the perovskite absorber from degradation.^32^ It is important to note that all these UV stability tests of the PSCs have been carried out using UV light source with wavelength <380 nm. PSCs exhibit >70% external quantum efficiency (EQE) around 400 nm,^20,33,34^ indicating their potential to exclusively harvest energy from the narrow near-ultraviolet (UV-A) region.

However, short wavelength near-UV (UV-A) light, also called black light, commonly used under indoor environments for ideal black lighting effects (e.g., in bars, pubs, aquariums, parties, clubs, body art studios, and as neon lights for Christmas and Halloween decorations) has not been comprehensively explored as a light source for energy harvesting despite the high photon energy than that of the white light. Importantly, these near-UV harvesting solar cells can find niche market for powering low power electronics (e.g., IoT sensors) located in environments with near-UV lighting, for example, in healthcare sectors,^35−37^ for bacterial disinfection,^38−40^ and in horticulture for the high yield plant growth.^41,42^

In the present study, as a proof-of-concept, we have fabricated and optimized PSCs with the commonly explored perovskite absorber CH_3_NH_3_PbI_3_ in a typical *n*–*i*–*p* device architecture FTO/SnO_2_/CH_3_NH_3_PbI_3_/Spiro-OMeTAD/Ag and studied their photovoltaic performance using commercially available near-UV indoor LED light [TBE Lighting, A60 UV LED, 395–400 nm, 9 W] at different illumination intensities in air [∼50% relative humidity (RH), ∼22 °C]. As the near-UV LED has the wavelength range above 380 nm and it lies in the visible region (380–700 nm), the intensity has been measured using an ILT 350 spectrometer both in lux and mW/cm^2^ (more details about the intensity measurement and the spectrum of the near-UV LED are given in the Experimental section in the Supporting Information). The term UV used throughout the manuscript refers to the near-UV region (395–400 nm) with the peak position at ∼399 nm. Importantly, a UV-stable configuration was realized by adapting the PCBM–BPhen interlayer^29^ between SnO_2_ and the perovskite layer, which not only enhanced the PCE and power output but the devices also retained 95.53% of the initial PCE after 24 h near-UV light exposure. This work opens up a new direction for PSCs to harvest energy from commonly used near-UV indoor LED lights to self-power microwatt energy devices.

## 2 Results and Discussion

**Figure 1:**
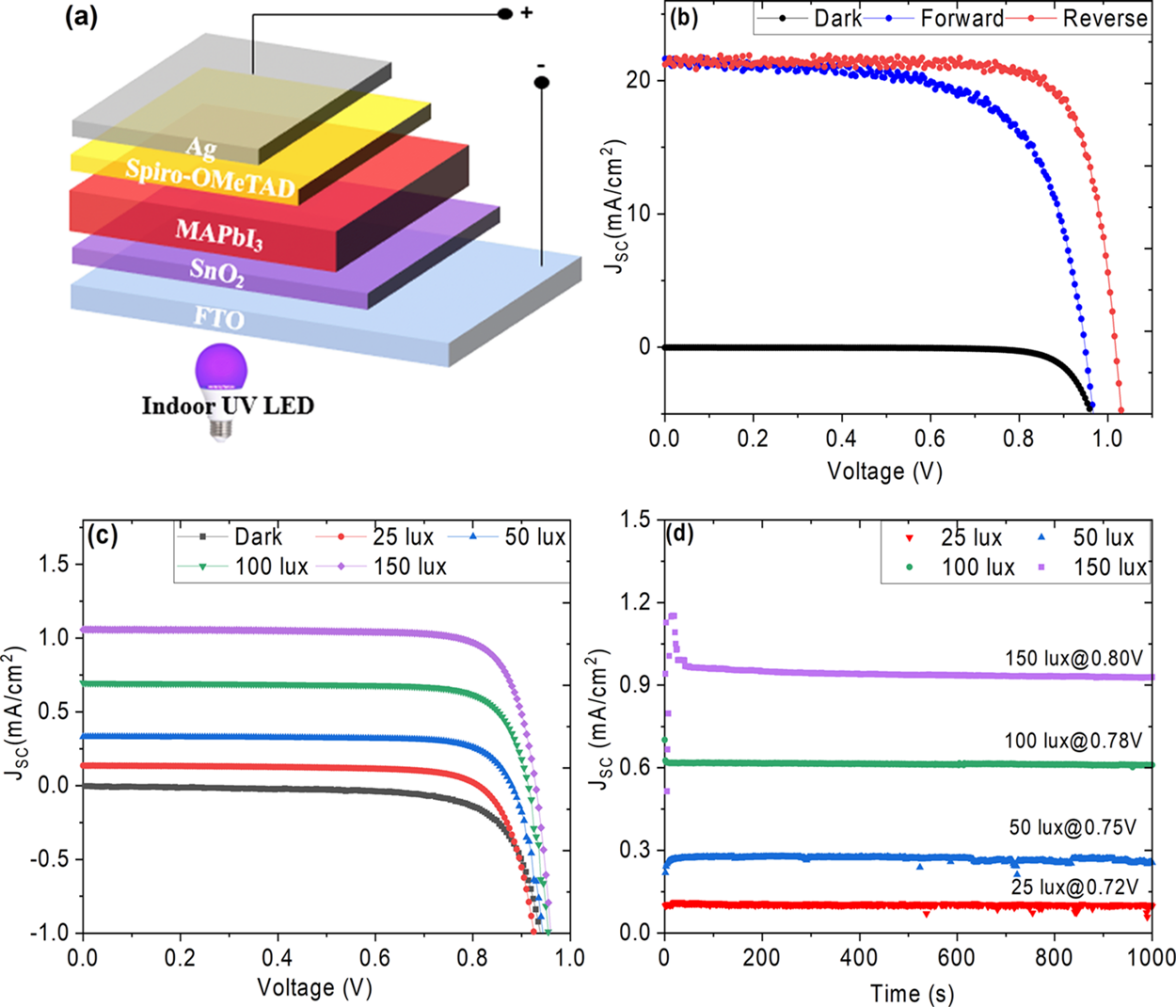
(a) Schematic of the perovskite solar cell device architecture, (b) J–V curves of the solar cell under 1 sun, (c) J–V curves of the solar cell under near-UV LED [9 W, 395–400 nm] at different illumination intensities (lux), and (d) stable current density of the solar cell biased at the maximum power point at different light UV intensities (lux).

PSCs were fabricated with the device architecture of FTO/SnO_2_/MAPbI_3_/Spiro-OMeTAD/Ag depicted in Figure 1 (a). The detailed device fabrication and characterizations can be found in the Experimental section in the Supporting Information. The PSCs were first tested under 1 sun condition, and the corresponding J–V plots under illumination (forward and reverse scan) and dark conditions are shown in Figure 1 (b). The J–V curve under reverse scan for 1 sun showed a PCE of 17.33% with a short circuit current density (*J*_SC_) of 21.41 mA/cm^2^, an open-circuit voltage (*V*_OC_) of 1.02 V, and a fill factor (FF) of 79.56%. The photovoltaic performance under near-UV light was studied using a low power near-UV LED [9 W 395–400 nm], generally used for indoor decoration. A dark box fitted with a UV LED lamp was used for the indoor light measurements (See Figure S1a, in the Supporting Information). The incident power of the UV LED was adjusted by varying the distance between the solar cell and lamp and measured using the ILT350 spectrophotometer having a measurement range of 380–780 nm. The ILT350 spectrophotometer recorded the near-UV LED source spectrum with the peak at ∼399 nm, as shown in Figure S1, which is consistent with the specification from the UV-LED manufacturer.

The maximum intensity for measuring the device performance was fixed at 150 lux, which corresponds to an incident power of 3.76 mW/cm^2^. It is worth noting that the incident power for indoor white LEDs at 500 lux is ∼0.150 mW/cm^2^, whereas for the 150 lux near-UV LED, it is 3.76 mW/cm^2^ because of high energy UV photons. This further implies that about 12,500 lux from white LED is required to get the equivalent intensity of near-UV LED used in this study at 150 lux.

**Figure 2:**
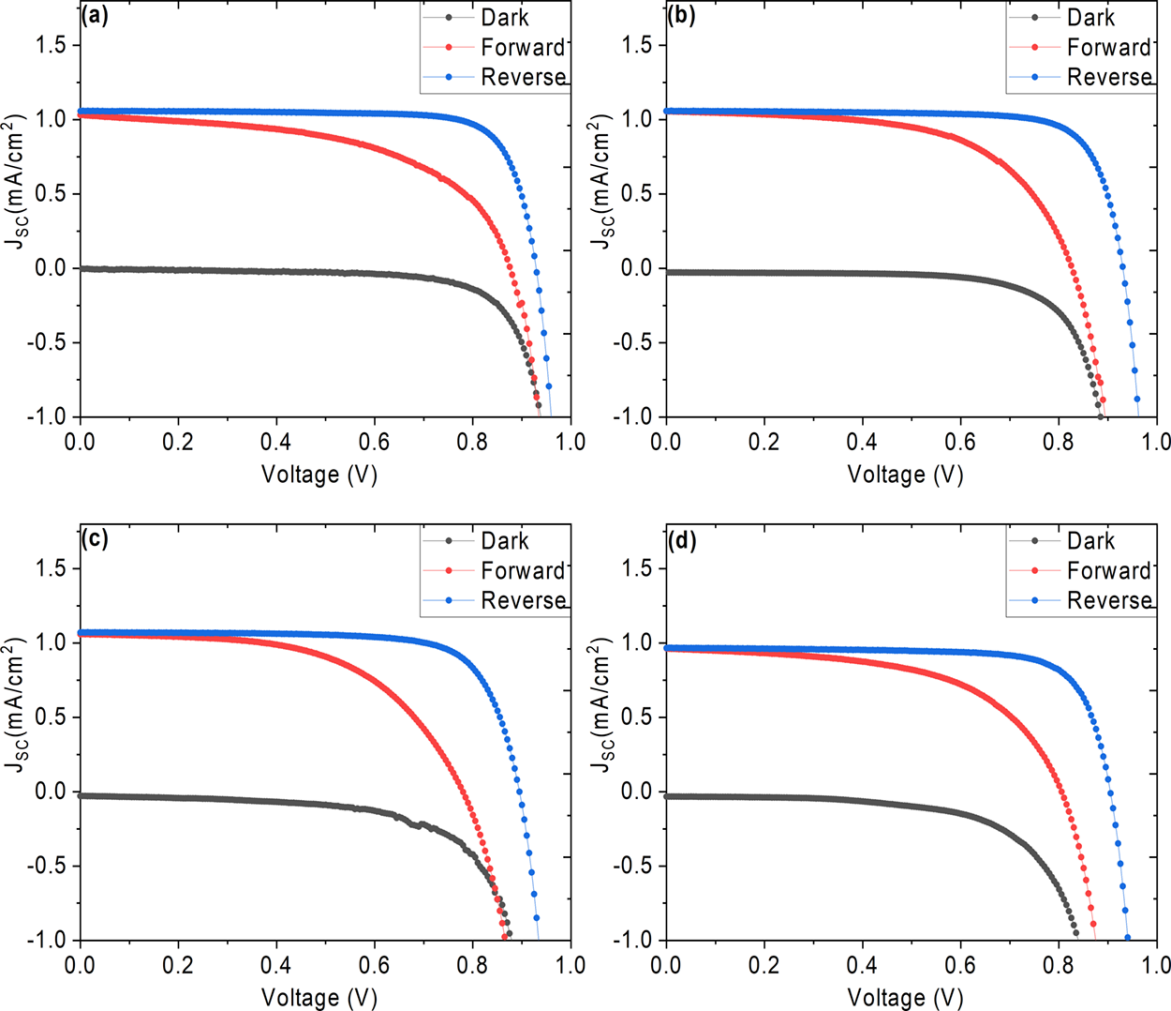
J–V curves solar cells measured under 150 lux near-UV light in air after being pre-treated to near-UV light for (a) 0 h, (b) 6 h, (c) 14 h and (d) 24 h.

It is interesting to note that the AM1.5G solar spectrum under 1 Sun (100 mW/cm^2^) has a near-UV intensity with an input power of 4.6 mW/cm^2^ at wavelengths below 400 nm. The J–V curves (reverse scan) of the PSC illuminated using the near-UV LED at different incident power are shown in Figure 1 (c) and the corresponding photovoltaic parameters are summarized in Table 1. At low illumination of 25 lux (0.63 mW/cm^2^), the device yielded a PCE of 11.68% with a power output of 70 μW/cm^2^, which increases further with increasing incident power. At high illumination of 150 lux (3.76 mW/cm^2^), the device achieved a champion PCE of 20.63% with *J*_SC_ of 1.06 mA/cm^2^, *V*_OC_ of 1.02 V, and a FF of 78.89%, resulting in a maximum power output of 775.86 μW/cm^2^. In general, *I*–*V* characteristics of the solar cells under visible indoor light are usually measured as a function of illumination (in lux) and the current density is observed to increase with the increase in the illumination intensity, as reported elsewhere.^9,43^ For near-UV light, even low illumination can generate high power as a result of high energy photons. It is interesting to note that even at 25 lux, the device delivers a power output of 70 μW/cm^2^, which can be sufficient to power up low power electronics, for example, IoT sensors.^44^ The J–V curves under dark and near-UV illumination (forward and reverse scans) are shown in Figure S2 and the photovoltaic parameters are summarized in Table S1. In order to understand the stable performance of the solar cells under indoor near-UV LED, the devices were biased at their maximum power point illuminated at different incident power continuously over a period of 1000 s in air (RH = ∼30 to 50%, *T* = 22 °C). The stable *J*_SC_ values of PSCs as a function of time are shown in Figure 1 (d). The devices showed stable performance for the entire maximum power point tracking without significant drop in *J*_SC_. However, it can be noted that for the illumination at low incident power, the *J*_SC_ stabilized from a low to higher value after certain time. On the other hand, burn-in decay was observed for devices illuminated under high incident power. It has been reported that the stable power output traces at maximum power point can be described with a double exponential decay function showing an initial rapid and a subsequent slower decay regime.^45,46^ The later decay is attributed to the degradation of one of the components in the device while the earlier is related to the halide migration within the perovskite as a result of the applied electric field. The devices illuminated at 100 and 150 lux at the maximum power point showed high *J*_SC_ upon illumination which then drops to stabilize the *J*_SC_ immediately. The burn-in loss at high incident power can be suppressed by implementing strategies reported for UV-stable PSCs.^29,47^ For example, the burn-in decay observed with the SnO_2_ electron transport layer was suppressed by introducing [6,6]-phenyl-C_61_-butyric acid methyl ester (PCBM) as an interlayer to block the photocatalytic effect of SnO_2_, and further introducing a small amount of bathophenanthroline (Bphen) molecule can largely stabilize the PCBM under UV light soaking.^29^ The box charts in Figure S3 show the statistical distribution of the photovoltaic parameters from 20 devices prepared in different batches, showing good consistency. PSCs tend to degrade under UV light as explored in a number of reports.^30,48,49^ In order to understand the indoor near-UV-light-induced degradation on the device performance, the devices were pretreated to near-UV LED light at 100 lux for 6, 14, and 24 h (termed as UV pretreated solar cells) in air. The J–V characteristics of the UV pretreated devices were then measured under 150 lux. The reverse and forward scan J–V plots under 150 lux black light along with dark curves for these UV pretreated solar cells are shown in Figure 2. The corresponding photovoltaic parameters are summarized in Tables 2 and S2, and the statistical distributions with box charts are shown in Figure S4. The devices UV pretreated for 6 h retained 97.18% of their maximum PCE while 14 h pretreated devices retained 92.38%. However, further increasing the UV pretreatment time to 24 h led to significant drop in PCE and the devices retained 84.1% of their maximum PCE. The fill factor is the predominant parameter that was influenced upon long UV exposures, which is consistent with previous report.^30^ The drop in the fill factor could be attributed to methyl ammonium group degradation upon UV light exposure^50,51^ leaving defects and trap states, which affect the photogenerated charge carrier transfer to respective selective contacts. Interface engineering in PSCs have proven to improve the device stability under UV light exposure,^52−54^ and we believe that those strategies would be applicable for highly stable indoor UV light-harvesting PSCs as well. For example, the ZnO-ZnS cascade electron transport layer in PSCs displayed improved UV stability by retaining 87% of its initial PCE over 500 h under UV radiation (365 nm, Spectroline SB-100P/FA) at RH ∼ 70%.^25^ Similarly, PSCs with TiO_2_/C_60_ bilayer retained 83% of its initial PCE after 312 h of UV radiation by eliminating negative charge accumulation at the perovskite/ETL interface.^55^

**Table 1:** Photovoltaic Performance of PSCs under 9 W, 395–400 nm near-UV LED at Different Incident Intensities

Intensity/P_in_ [Lux/(mW.cm^–2^)]	PCE (%)	FF (%)	*J*_SC_ (mA/cm^2^)	*V*_OC_ (V)	MP (μW/cm^2^)
25/0.63	11.68	64.92	0.14	0.8	70.00
50/1.37	16.30	76.55	0.33	0.88	223.27
100/2.78	17.87	77.99	0.70	0.91	495.71
150/3.76	20.63	78.89	1.06	0.93	775.86
1 sun/100	17.33	79.56	21.41	1.02	17,330,000

**Table 2:** Photovoltaic Parameters of the PSCs Measured under Incident Intensity of 150 lux after Pretreatment to near-UV Light [100 lux] for Various Time Periods

UV pretreatment time (h)	PCE (%)	FF (%)	*J*_SC_ (mA/cm^2^)	*V*_OC_ (V)	MP (μW/cm^2^)
0	20.63	78.89	1.06	0.93	775.90
6	20.05	77.30	1.06	0.92	754.03
14	19.06	74.70	1.07	0.90	716.58
24	17.77	76.32	0.96	0.90	666.83

**Figure 3:**
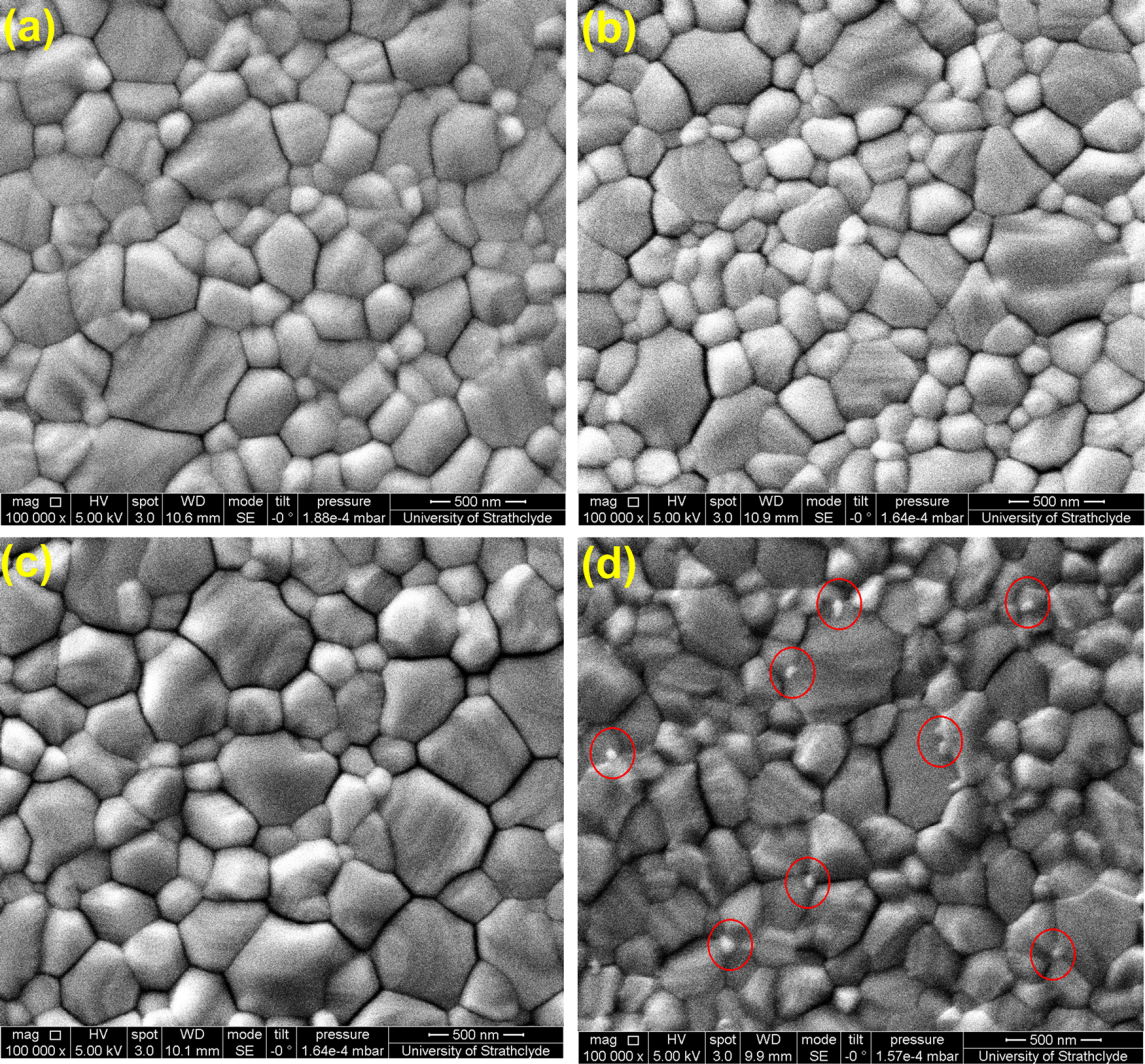
Top-view SEM images of the perovskite films pretreated with near-UV light (100 lux) for (a) 0 h, (b) 6 h, (c) 14 h, and (d) 24 h.

**Figure 4:**
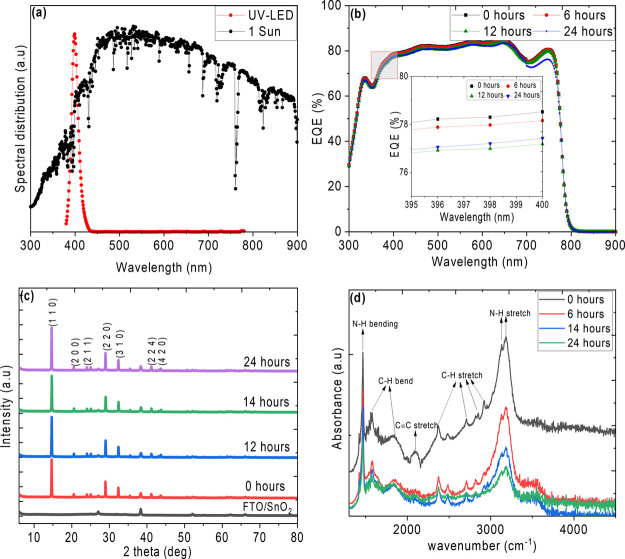
(a) Relative spectral distribution of AM1.5G 1 sun and 9 W UV LED, (b) EQE spectra of the solar cells UV pretreated for different time durations (inset shows an enlarged view corresponding to the wavelength region of the UV LED source), (c) XRD pattern, and (d) ATR-FTIR spectra of perovskite films.

In order to explore the effects of UV exposure on the morphology of perovskite films deposited on the SnO_2_-coated FTO substrate, we carried out scanning electron microscopy (SEM) analysis, and the top-view SEM images of the films are presented in Figure 3. The unexposed and 6 h UV pretreated films show densely packed film and larger grains with the size of about 500 nm, as seen in Figure 3 (a) and (b), which agrees with the photovoltaic parameters summarized in Table 2. Further increasing the exposure time to 14 h led to more visible grain boundaries, as seen in Figure 3 (c), indicating the initial stage of degradation. The films exposed to 24 h shows some white regions (highlighted using red circles) appearing at the grain boundaries, with poor contrast as seen in SEM Figure 3 (d), which we speculate to methyl ammonium lead iodide degradation and accumulation of lead iodide at the grain boundaries. It was difficult to do elemental mapping on this region alone to get more quantitative analysis. Furthermore, we attribute that these white regions possibly reduce the charge carrier extraction at the interfaces as even minor defects are detrimental to indoor energy harvesting due to less electron–hole pair generation, which agrees with the photovoltaic parameters summarized in Table 2. However, these features do not play a significant role in the optical absorption and structural properties as discussed. Similar observations for CH_3_NH_3_PbI_3_ films have been reported, in which the perovskite films were UV-pretreated (PHILIPS UV-A lamps emitting at 350–400 nm, 10 mW/cm^2^) for different time periods and observed surface irregularities with high roughness as a result of UV-light-induced degradation.^56^ External quantum efficiency (EQE) measurements were carried out for the UV pretreated solar cells to analyze the photon energy conversion efficiency at the wavelength corresponding to the indoor near-UV LED source. Figure 4 (a) shows the relative spectral distribution of the AM 1.5G 1 sun and 9 W 395–400 nm near-UV LED used in the present study. Figure 4 (b) shows the EQE spectra of UV-pretreated PSCs, and the inset shows an enlarged view of the spectrum in the wavelength range of the UV source used (395–400 nm). The unexposed device shows ∼78% EQE while the 24 h UV pre-treated device holds ∼77% EQE. Figure 4 (c) shows the X-ray diffractograms of the perovskite films deposited on SnO_2_-coated FTO substrates after UV exposure for various times. There are no significant differences observed in the XRD patterns of the indoor UV pretreated films. All the films show a dominant diffraction peak at 14.7° corresponding to the (1 1 0) plane of the tetragonal phase along with minor peaks at higher angles. It is interesting to note that the peak positions of the unexposed film remain unchanged after prolonged UV exposure, indicating that the perovskite phase is stable at these near-UV intensities for 24 h or the changes are not discernible in the XRD which can show only features corresponding to any new phase if it is >10%. We note that the decrease in photovoltaic performance from Figure 2 could not be correlated based on the XRD analysis as the defects generated in the perovskite films due to prolonged indoor near-UV LED exposure is minimal, and the white regions observed from SEM for 24 h exposed film does not influence the crystalline phase. To understand the potential change of CH_3_NH_3_ in the UV pretreated perovskite films, Fourier-transform infrared (FTIR) spectra were evaluated in attenuated total reflectance (ATR) mode. The ATR-FTIR spectra of the perovskite films shows the methyl and ammonium functional groups with bending and stretching modes, as shown in Figure 4 (d). Upon UV pretreatment, the intensity of the vibrational modes decreases even for 6 h treatment. However, the N–H bands at 1465, 3124, and 3197 cm^–1^ diminish significantly than those of the C–H bands, indicating that deprotonation of NH_3_ is the main step in the degradation process. Similar observations of NH_3_ deprotonation in CH_3_NH_3_PbI_3_ films were reported as a result of light-induced degradation.^50^ The optical absorption spectra of UV pretreated films prepared on glass substrates are shown in the inset of Figure S5. All the films show characteristic absorption onset at around 790 nm corresponding to the CH_3_NH_3_PbI_3_ band gap (1.56 eV).

**Figure 5:**
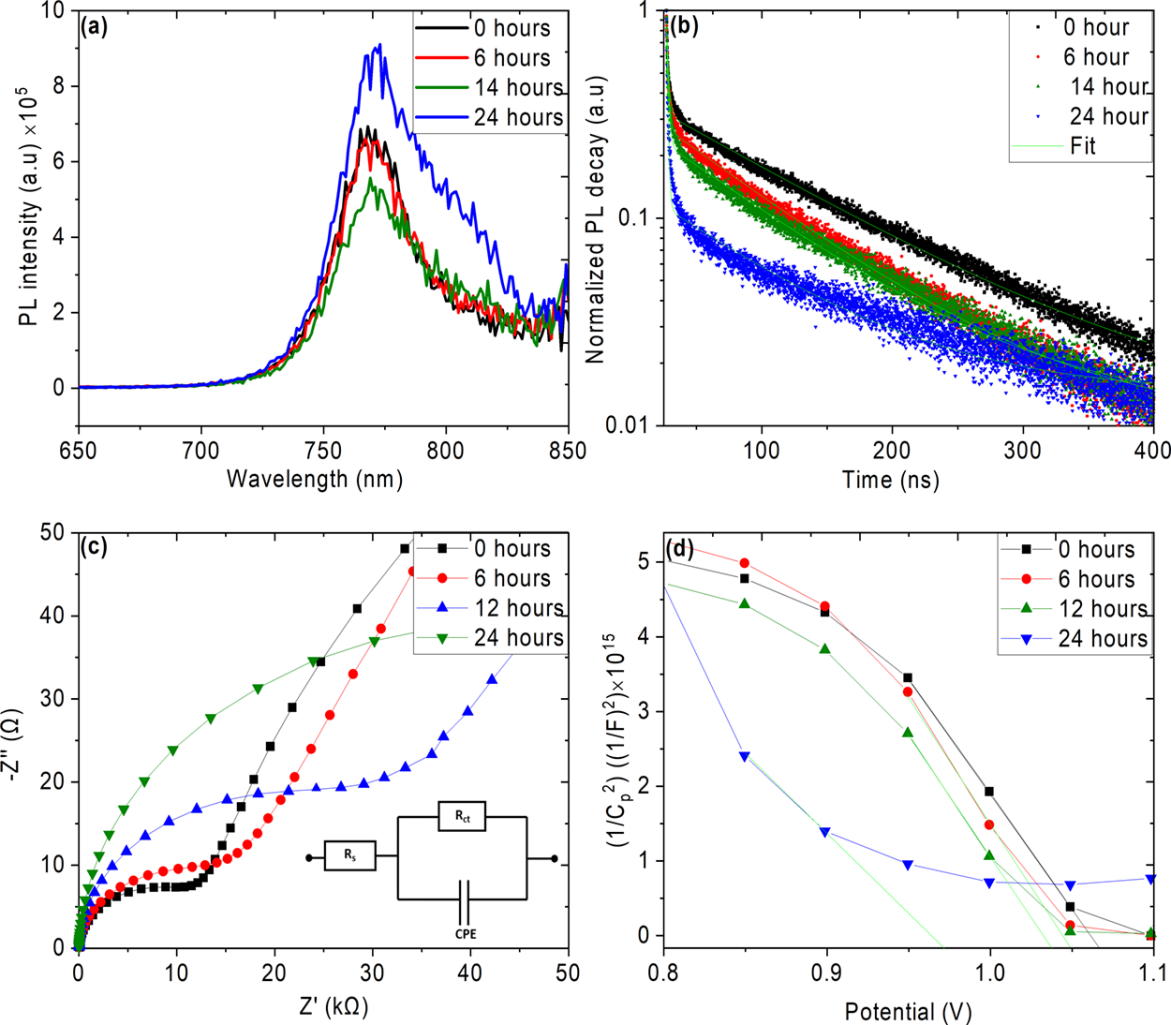
(a) PL spectra and (b) TRPL spectra of UV-pretreated CH_3_NH_3_PbI_3_ films, (c) Nyquist (with equivalent circuit used for fitting given in the inset), and (d) M–S plots of the PSCs pretreated with UV light for different time periods.

The photoluminescence (PL) and time resolved photoluminescence (TRPL) spectroscopy are useful techniques that can be used to investigate the change in carrier dynamics and recombination from minor defects and trap states. The PL spectra of near-UV pretreated perovskite films deposited on glass are shown in Figure 5 (a). The unexposed film shows characteristic PL emission at around 768 nm associated with the band to band transition. The PL emission peak position remains the same for the 6 and 14 h UV pretreated films, although with reduced intensity as compared to untreated samples. Further increasing the UV pretreatment time to 24 h redshifts the PL peaks to 771 nm with a tail at high wavelength at 802 nm (Figure S6). This redshift could be attributed to intrinsic defects and trap states caused by degradation by methyl group decomposition because of UV exposure leaving PbI_2_-rich domains, which is the predominant factor for the drop in the photovoltaic performance of the devices discussed in Figure 2. Similar PL redshift with enhanced intensity was observed in PbI_2_-rich MAPbI_3_ films.^57^ The TRPL spectra of the films deposited on the glass substrates are shown in Figure 5 (b) and the curves were fitted with two exponential functions with one decay component associated with the fast surface radiative recombination decay (τ_1_) and another one with the slow bulk radiative recombination decay (τ_2_).^58^ The average lifetime of the films was calculated using the relation (A_1_τ_1_ + A_2_τ_2_)/(A_1_ + A_2_),^59,60^ where τ and A are the decay and amplitude components, respectively. The decay time and amplitude components determined from the fittings are summarized in Table S3. The average decay time of charge carriers in the untreated film is 118.16 ns (τ_1_ = 1.52 ns and τ_2_ = 118.25 ns), indicating that the photogenerated charge carriers can be efficiently transferred to the respective charge selective contact and thus interface recombination can be suppressed. Upon UV pretreatment, the average decay time reduced to 28 ns (τ_1_ = 0.96 ns and τ_2_ = 92.01 ns) for 24 h pretreated film, which can be related to interface recombination, which in turn results in poor photovoltaic performance. We note that the average decay time of the UV pretreated sample is very low despite high τ_2_ because of the poor A_2_, as shown in Table S3. The electrochemical impedance spectroscopy (EIS) measurements were carried out to further investigate the charge carrier transport process in UV-pretreated PSCs. Figure 5 (c) shows the Nyquist plots of the near-UV-exposed solar cells under dark at 0 V potential. The first arc at a high frequency region is usually related to the carrier transport resistance or contact resistance (*R*_ct_) at the interface between the perovskite/ETL in the device while the second arc at lower frequency is usually attributed to the charge recombination resistance within the perovskite film.^61,62^ From the Nyquist plot, *R*_ct_ can be extracted by fitting the plot using the equivalent circuit model shown in the inset of Figure 5 (c). The numerical fitting reveals *R*_ct_ of 14.48, 22.64, 38.76, and 73.69 kΩ respectively, for 0, 6, 14, and 24 h UV-pretreated PSCs. The small *R*_ct_ determines efficient charge transfer from the perovskite layer to the electron transport layer. The high *R*_ct_ for 24 h UV-treated PSCs reveals that the interface defects are predominant with UV exposure, which causes charge accumulation at the CH_3_NH_3_PbI_3_/SnO_2_ interface. EIS is further used to analyze the voltage modulation and characterize the built-in potential (*V*_bi_) of UV pretreated PSCs using Mott–Schottky (M–S) relationship, as shown in Figure 5 (d). The *V*_bi_ is defined by the intersection of the 1/*C*^2^ curve and horizontal bias axis, which probes into charge accumulation at the interface between the MAPbI_3_ and SnO_2_ that affect the potential barrier. The *V*_bi_ of the unexposed device showed 1.06 V while the 6, 14, and 24 h UV pretreated devices show 1.04, 1.02, and 0.97 V, respectively. The high *V*_bi_ indicates fast charge collection and reduced carrier accumulation,^63^ and decrease in *V*_bi_ can be attributed to the increase in trap state densities as a result of bulk and surface degradation.

**Figure 6:**
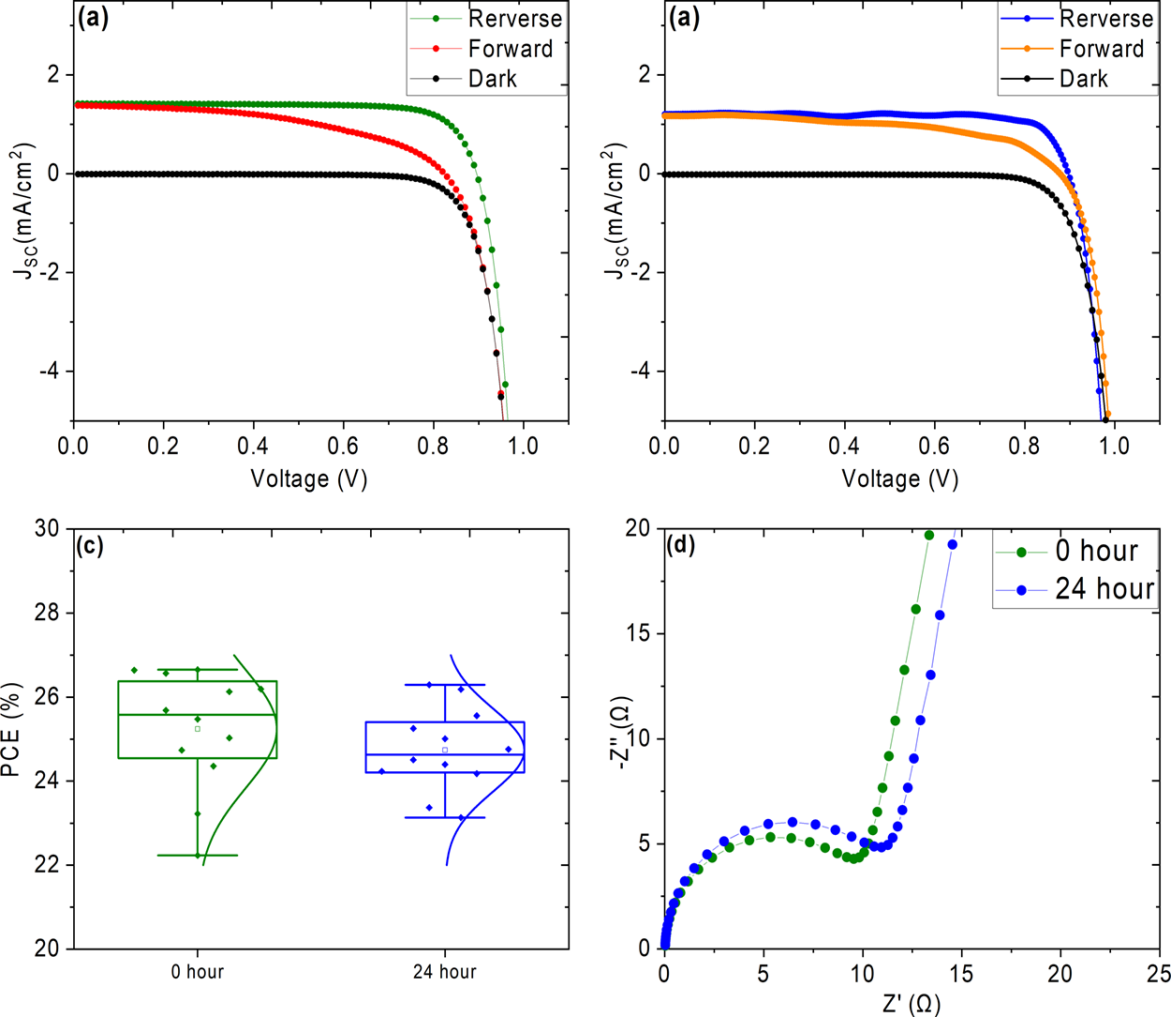
J–V curves of (a) 0 h and (b) 24 h UV-pretreated PSCs with the SnO_2_/PCBM–Bphen ETL, (c) box chart PCE of the solar cells, and (d) Nyquist plot of PSCs measured under near-UV illumination of 150 lux.

**Figure 7:**
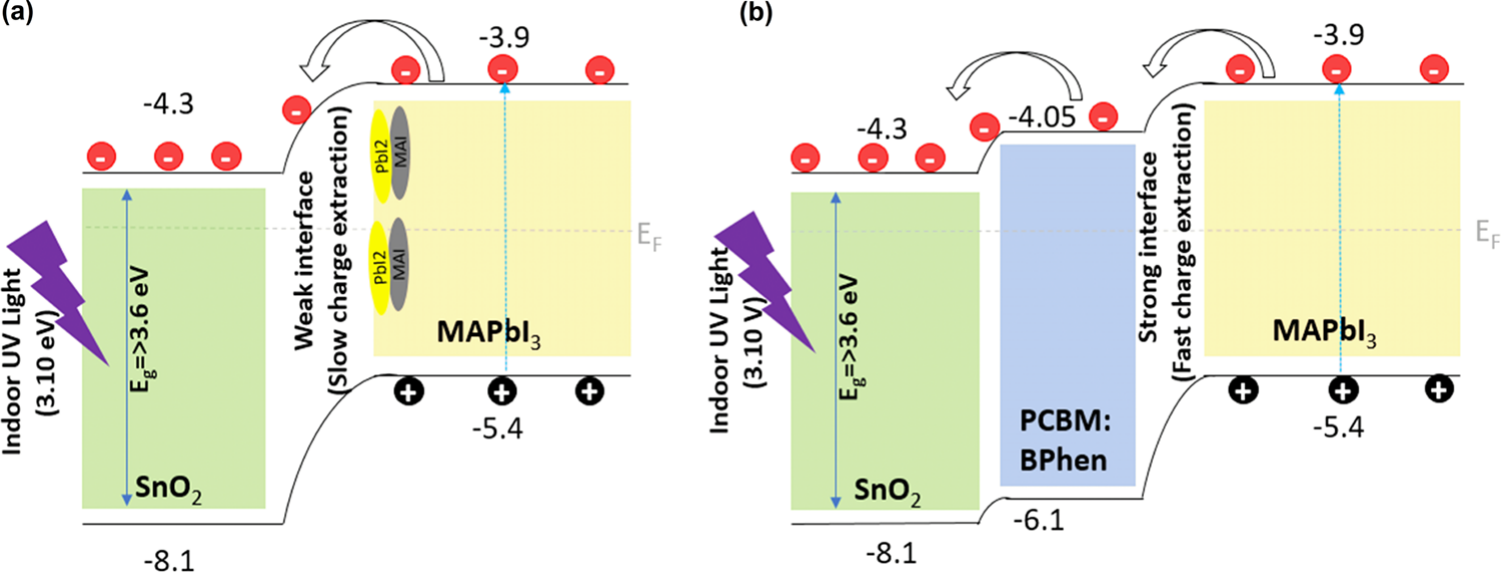
Schematic representation of the band diagram and indoor UV-light-generated charge transfer mechanism of PSCs with negligible photocatalysis reaction from SnO_2_.

Finally, we adapted the PCBM–BPhen interlayer between the SnO_2_ and perovskite film to fabricate UV-stable configuration.^29^ This is to show that the present proof-of-concept black light-harvesting devices can be made more efficient with the UV-stable configuration. The J–V curves of a champion device under 1 sun illumination, which delivered a PCE of 18.29% (reverse scan), are shown in Figure S7. The photovoltaic parameters are summarized in Table S4, and the box charts for the statistics are shown in Figure S8. The devices were pretreated under near-UV LED light similar to SnO_2_ ETL-based devices discussed earlier. The J–V curves of 0 and 24 h UV light pretreated solar cells under near-UV illumination are shown in Figure 6 (a) and (b) respectively, and the resulting photovoltaic parameters are summarized in Table 3. The as-fabricated champion device delivered an enhanced PCE of 26.19% and a power output of 991.21 μW/cm^2^ as a result of enhanced electron transfer from perovskite to the ETL with the interlayer.^29,60^ The PCBM–Bphen interlayer prevented UV-induced degradation at the interface and retained 95.53% of the initial PCE when pretreated to 24 h. The improved hysteresis upon UV light pretreatment could be correlated to light-induced defect passivation.^64^Figure 6 (c) shows the PCE box chart statistics of the devices. The Nyquist plots of 0 and 24 h UV-pretreated solar cells are shown in Figure 6 (d). In contrast to the SnO_2_ ETL only devices discussed in Figure 5 (c), the PCBM–BPhen interlayer between the ETL and perovskite maintains the *R*_ct_ of UV-pretreated devices close to that of the untreated devices. The *R*_ct_ values of 0 and 24 h UV-pretreated devices are 15.90 and 19.94 kΩ, respectively, which could be correlated to obtain photovoltaic performance without significant loss. Based on the device performances, the mechanism for the UV-stable configuration is shown as a schematic in Figure 7. It is important to note that the photocatalytic reaction by SnO_2_ is negligible as the indoor UV light wavelength is smaller (∼3.10 eV) than the band gap of SnO_2_ (∼3.6 eV). Several strategies have been reported to reduce the UV-induced degradation of the PSCs with organic interfacial layers like PCBM on metal oxides which enhance the device efficiency with improved long-term device stability under simulated full-spectrum sunlight.^29,65,66^ Such an organic interlayer anchors the functional groups between the ETL and perovskite more strongly to reduce the oxygen vacancy defects at the interface which would otherwise cause the interface instability.^67,68^ We attribute that the enhanced charge extraction and stability of the interface-modified devices is likely from the chemical interface stability of the perovskite/PCBM–BPhen where deep-level defects of SnO_2_ were passivated by the organic interlayer.^65^ The determined shunt resistance and series resistance of the devices based on SnO_2_ and SnO_2_/PCBM–Bphen ETL are summarized in Table S5. The shunt resistance increased more than fivefold after the PCBM–Bphen interlayer, which is beneficial for enhanced charge extraction. Furthermore, the surface morphology and crystallinity of perovskite film can be different depending on the underlying layer which could improve the interface stability;^66^ however, it is beyond the scope of this present work. As the photocatalytic reaction from SnO_2_ is negligible, we believe that PSCs with defect-free interface can lead to highly efficient and stable devices under indoor UV light.

**Table 3:** Photovoltaic Parameters of UV Pretreated PSCs with the PCBM–BPhen Interface Layer Measured under near-UV Illumination of 150 Lux

UV pretreatment time (h)	Scan	PCE (%)	FF (%)	*J*_SC_ (mA/cm^2^)	*V*_OC_ (V)	HI index (%)
0 h	R	26.19	77.56	1.42	0.90	40.09
	F	14.38	47.41	1.38	0.82	
24 h	R	25.02	84.39	1.25	0.89	35.10
	F	16.24	59.69	1.18	0.87	

In general, the following strategies are required to be considered for designing efficient indoor UV light-harvesting solar cell architectures. In contrast to solar cells operated under 1 sun condition, electrode requirements for indoor visible harvesting solar cells will have some flexibility on their conductivity and sheet resistance, provided the shunt resistance of the device is large. This is because series resistance does not have a drastic effect on device performance under indoor visible light operation, and thus, expensive TCO or metal electrodes can be replaced with less conducting electrodes. Under indoor UV conditions on the other hand, the generated photocurrent is relatively larger as compared to that under indoor visible light, and thus, the influence of *R*_s_ is relatively larger because of the higher electrical voltage drop on the *R*_s_. On the other hand, the influence of the *R*_sh_ also plays an important role if the irradiance becomes very weak, and the shunting current may be comparable to that of the photocurrent. Thus, low *R*_sh_ causes severe power losses by trapping most of the photogenerated charge carriers through shunting paths. Additionally, the degree of spectral overlap between the emission spectra of indoor UV lights and the band gap of the photoactive layer will play a critical role in generating photocurrent and thus to yield high PCE. Furthermore, the larger band gap leads to increase in the open circuit voltage of a solar cell, which is better for achieving highly efficient devices. Thus, the UV-harvesting solar cells should consider a device design with low series resistance and high shunt resistance along with an absorber with the band gap matching the UV spectrum, which can be achieved via interfacial and compositional engineering.

## 3 Conclusions

In summary, we have demonstrated that PSCs designed for outdoor conditions [with device architecture FTO/SnO_2_/CH_3_NH_3_PbI_3_/Spiro-OMeTAD/Ag] can be effectively used to harvest energy from the near-UV indoor LEDs (395–400 nm) that are widely used as indoor decoration black lights. The champion device delivered a PCE of 20.63% at 150 lux with the maximum power output of 775 μW/cm^2^ and showed a stable current density over a period of 1000 s at the maximum power point. The devices aged under continues near-UV illumination for 24 h retained >84% of its initial PCE. The drop in performance upon near-UV exposure for different durations has been comprehensively characterized. Moreover, UV-stable solar cells fabricated with the PCBM–BPhen interfacial layer [with device architecture FTO/SnO_2_/PCBM-BPhen/CH_3_NH_3_PbI_3_/Spiro-OMeTAD/Ag] maintained the contact resistance between the ETL and the perovskite layer under continuous UV illumination for 24 h and retained 95.53% of their initial PCE of 26.19%. This work opens up a new direction for advancement in the practical deployment of PSCs for indoor near-UV light energy harvesting and thus is beneficial for powering modern electronics integrated with sensors and IoTs located in UV environments such as health care, horticulture, and places with near-UV black light decorations.

## Supporting Information Available

Additional J–V curves; photovoltaic parameter table; statistical distribution of the solar cell parameters; optical absorption spectra; and PL spectra with fitting (PDF)
