# Supplementary material for: Near-Ultraviolet Indoor Black Light-Harvesting Perovskite Solar Cells
Source: ACS Appl Energy Mater. 2022 Nov 17;5(12):14669–79. doi: 10.1021/acsaem.2c01560 (PMC9795417; doi:10.1021/acsaem.2c01560)
Supplement: Supplementary file 1 — ae2c01560_si_001.pdf [file ae2c01560_si_001.pdf]

Supporting Information

Near-UV Indoor Black Light Harvesting Perovskite Solar Cells

Arivazhagan Valluvar Oli[a], Zinuo Li[b], Yu Chen[b] and Aruna Ivaturi[a]*

[a] Smart Materials Research and Device Technology (SMaRDT) Group, Department of Pure and Applied Chemistry, University of Strathclyde, Thomas Graham Building, Glasgow, G1 1XL, United Kingdom.

[b] Department of Physics, University of Strathclyde, Glasgow, G4 0RE, United Kingdom.

Corresponding Author

*aruna.ivaturi@strath.ac.uk

Experimental and characterization techniques

Materials

All the materials used in this work were purchased from commercial suppliers, and used as received. These include Lead (II) iodide (PbI_2, 99.99 %, TCI), Methylammonium iodide (MAI, >99.99%, GreatCell Solar), Tin (IV) oxide [15% in H_2O colloidal dispersion] (Alfa Aesar), Spiro-OMeTAD [>99.5%, (HPLC), Luminescence Technical Corp.], Bis(trifluoromethane)sulfonimide lithium salt (LiTFSI, Sigma-Aldrich), N,N-Dimethyl formamide (DMF, 99.8%, Acros organics), Dimethyl sulfoxide (DMSO, 99.7+%, Acros organics), Chlorobenzene (99.8% Acros organics), 1-Butanol (99.8%, Sigma-Adrich), 4-tert butylpyridine (98 %,Sigma-Adrich), Zn powder (99.8+ %, Acros organics), PCBM (>99.5%, Luminescence Technical Corp), BPhen (Sublimed >99.0%, Ossila), Hellmanex III (Sigma-Aldrich), Acetone (VWR chemicals), 2-propanol (VWR chemicals), Silver pellet (99.99%, Kurt. J. Lesker) and TEC 15 FTO substrates (15 Ω/sq, NSG Pilkington).

Device fabrication

For the fabrication of the solar cells, FTO substrates were patterned using Zn powder and 4M HCl. The patterned substrates were cleaned using brush and 2% Hellmanex solution (in Deionised water) followed by ultrasonication in acetone and 2-Propanol, for 15 min each. The substrates were then treated with oxygen plasma for 10 min. For depositing the electron transport layer, 70 μl of SnO_2 colloidal solution (1:5 v/v in water) was spin-coated on the cleaned FTO substrates for 30 seconds at 3000 rpm followed by annealing for 30 min at 150°C in air. The SnO_2 coated substrates were then treated with oxygen plasma for 10 minutes before transferring into the nitrogen filled glovebox for depositing the next layers. $CH_3NH_3PbI_3$ ($MAPbI_3$) precursor solution was prepared by mixing 642 mg of PbI_2 and 222 mg of MAI in 4:1 DMF:DMSO solvent. The precursor solution was spin coated on the SnO_2 coated substrate at 1000 rpm for 5 seconds, 2000 rpm for 10 seconds and 5000 rpm for 20 seconds. Chlorobenzene (200 μl) antisolvent

was dropped 15 seconds before the end of the spin cycle. The substrates were then annealed at 100 °C for 10 minutes to obtain the $MAPbI_3$ films. For PCBM:BPhen interlayer between SnO_2 and MAPI, 12.5mg PCBM was dissolved in 1 ml chlorobenzene and 200 µl Bphen (0.5 mg in 1 ml chlorobenzene) was added into it. The precursor was stirred overnight and spin coated on SnO_2 coated substrate at 3000 RPM for 30 seconds followed by annealing at 100 °C for 10 minutes. To deposit hole transport layer (HTL), Spiro-OMeATD precursor consisting of 72.5 mg/ml chlorobenzene with 17.5 µl of LiTFSI (520 mg/ml of 1-butanol) and 27.5 µl 4-tert butylpyridine was spin coated on $MAPbI_3$ layer at 3000 rpm for 30 seconds. Finally, to complete the device, silver electrodes (100 nm) were thermally evaporated (in 5×10^{-7} mbar vacuum) through a shadow mask.

UV LED source and intensity measurements

A commercially available near-UV LED light [TBE Lighting, A60 UV LED, 395-400 nm, 9W], generally used as an indoor decorating lamp, was used as the indoor light source. As the near-UV LED used in the present study emits within the visible region (380-700 nm), it was possible to measure the intensity in terms of lux. A dark box fitted with the near-UV LED lamp was used for the intensity and solar cell characterizations. A photospectrometer (model ILT 350, with measurement range of 380-780 nm) was used to measure the light intensity. The intensity and the irradiance of the near-UV LED could be directly read from the spectrophotometer. The spectrophotometer recorded the near-UV LED source spectrum with the peak at ~399 nm which is in consistent with the UV-LED specification from the manufacturer. The term UV used throughout manuscript and supporting information represent near-UV region (395-400 nm) with the peak position at ~399 nm.

UV pre-treatment of $MAPbI_3$ films and solar cells

For the UV degradation studies, $MAPbI_3$ films and solar cells were exposed (referred to as near-UV pre-treatment) to near-UV light [TBE Lighting, A60 UV LED, 395-400 nm, 9W] at 100 lux (2.78 $mWcm^{-2}$) for different time durations. All the samples were kept under ambient condition (RH:40-45 % and temperature of 20-24 ℃) for 24 hours irrespective of the near-UV pre-treatment duration. This is to guarantee same environmental impact on all the samples/devices.

Characterization

The surface morphology of $MAPbI_3$ films were imaged using the field-emission scanning electron microscope (FE-SEM, FEI Quanta 250 FEG). The optical absorption of the films was measured using UV-Vis spectroscopy (UNICAM UV-300). Fourier transform infrared (FTIR) spectroscopic measurements were carried out in attenuated total reflection mode (Agilent 0147A). The XRD measurements were performed at room temperature on a Bruker D2 Phaser system using monochromatic CuK_α radiation with a wavelength of 1.5406 Å. The samples were scanned in the range 5-80° with an increment of 0.04° on the 2θ scale. The substrates were set to a rotation speed of 8 rpm throughout the measurement. Photoluminescence (PL) measurements were done using Horiba Fluorolog 3-22 with xenon lamp at an excitation wavelength of 503 nm. TRPL measurements were done using Horiba Deltaflex TCSPC lifetime fluorometer with 503 nm laser source. The PL decay time was determined by fitting the curve using Origin pro software. The Electrochemical impedance spectroscopic measurements were performed using AUTOLAB PGSTAT 302N and FRA32M. External quantum efficiency (EQE) spectrum was taken using Bentham (PVE300) in transformer mode. Silicon reference diode was used for calibration.

Current density-Voltage (J-V) characterizations

The current-voltage measurements were carried out using Ossila Solar Cells I-V test system. For 1 sun measurement, the solar spectrum at AM 1.5 was simulated with a Xenon lamp and filters (Oriel Sol 1A, class ABB, 94021A) with the measured intensity at 100 mW cm^{-2}. The illumination intensity was calibrated using reference Si solar cell (Newport, 91150V). For indoor near-UV LED, a low power (9W) A60 UV LED lamp (TBE lighting) with the wavelength of 395-400 nm was used as a source. ILT350 spectrophotometer was used to measure the light intensity, incident power, and related spectral distribution of the LED light. The current density versus voltage (J-V) curves of the resulting devices were acquired in both forward-scan (FS; -0.2 to1.2 V) and reverse scan (RS: 1.2 to -0.2) with the scan speed of ~80 mV/s. The active area of the devices is 0.026 cm^2 determined by the standard aperture mask.

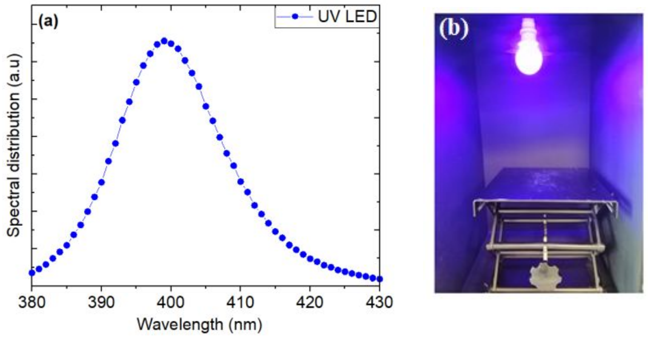

Figure S1: a) Spectrum of UV LED measured using ILT 350 and b) the indoor 9W UV LED source.

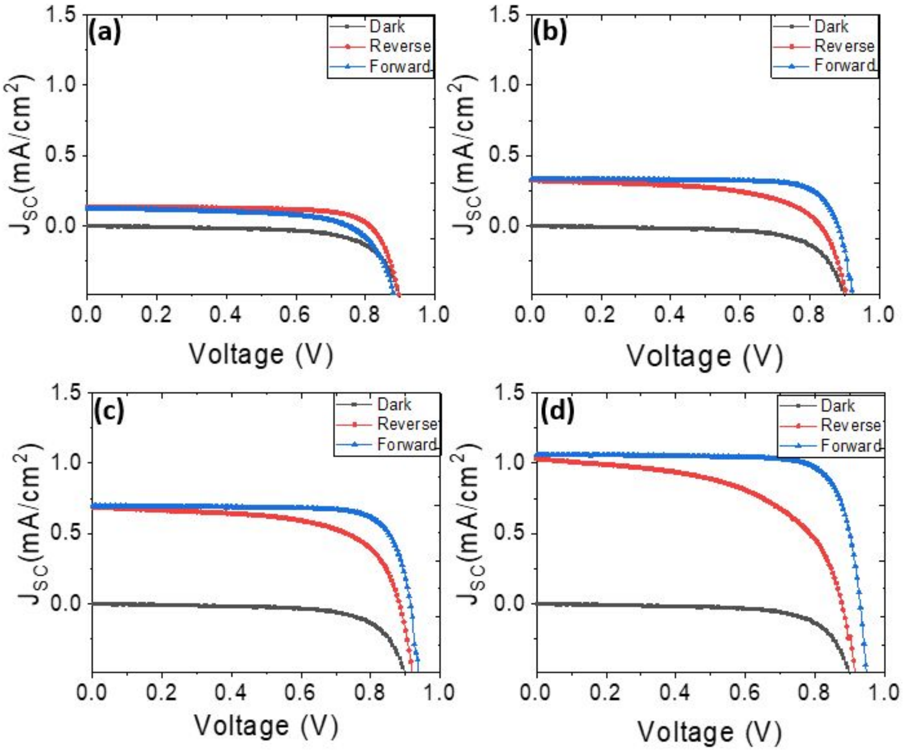

Figure S2. J-V curves (reverse and forward scans) of the perovskite solar cells [$FTO/SnO_2/MAPbI_3$/Spiro-MeOTAD/Ag] measured under a) 25 lux, b) 50 lux, c) 100 lux and d) 150 lux indoor near-UV light.

Table S1: Photovoltaic parameters of the solar cells [$FTO/SnO_2/MAPbI_3$/Spiro-MeOTAD/Ag] under different UV illumination intensity.

**Lux / P_{in} (mW/cm^2)**	**Scan**	**PCE (%)**	**FF (%)**	**J_{sc} (mA/cm^2)**	**V_{oc} (V)**	**MP ($\mu W/cm^2$)**	**Hysteresis index (%)**
**25 / 0.63**	F	7.13	49.95	0.12	0.8	50.00	38.95
	R	11.68	64.92	0.14	0.8	70.00	
**50 / 1.37**	F	10.66	54.50	0.32	0.83	146.10	35.25
	R	16.30	76.55	0.33	0.88	223.27	
**100 / 2.78**	F	13.34	61.12	0.69	0.88	371.17	25.35
	R	17.87	77.99	0.70	0.91	495.71	
**150 / 3.76**	F	13.00	54.04	1.03	0.88	488.67	36.98
	R	20.63	78.89	1.06	0.93	775.86	

The hysteresis index (HI) of the perovskite solar cells is calculated using the relation

$$HI=PCE_{reverse}-PCE_{forward} / PCE_{reverse.}$$

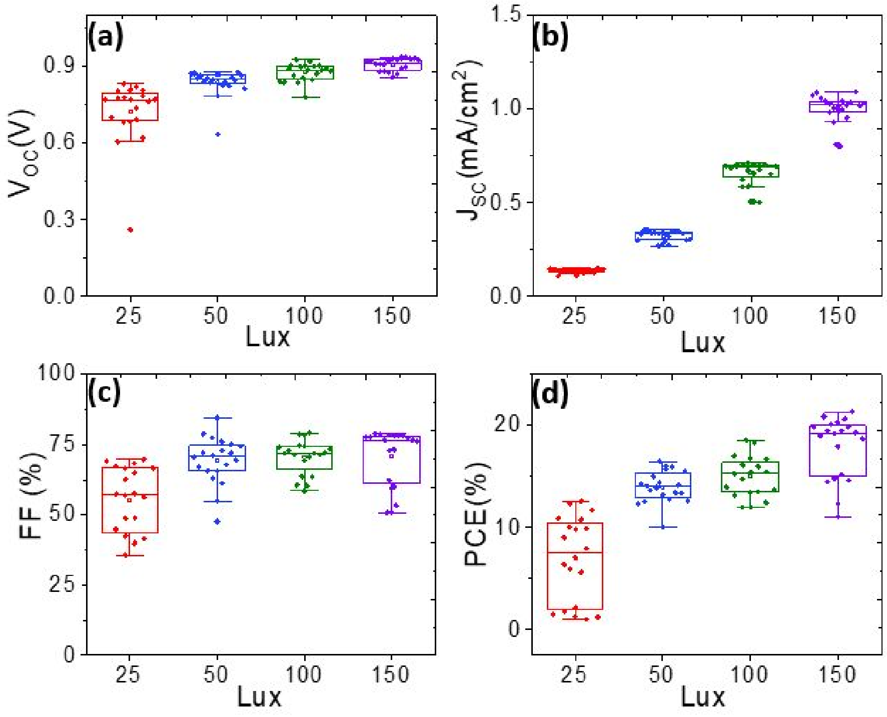

Figure S3. Statistical distributions of the photovoltaic metrics for 20 devices [FTO/SnO_2/$MAPbI_3$/Spiro-MeOTAD/Ag] measured under different incident illumination intensity.

Table S2. Photovoltaic parameters of the solar cells [FTO/SnO_2/$MAPbI_3$/Spiro-MeOTAD/Ag] under near-UV illumination (150 lux), measured after near-UV (100 lux) pre-treatment for different time durations.

UV Exposure time (hours)	Scan	PCE (%)	FF (%)	J_{SC} (mA/cm^2)	V_{OC} (V)	MP (μW/cm^2)	Hysteresis index (%)
**0**	F	13.01	54.04	1.06	0.93	488.70	36.93
	R	20.63	78.89	1.06	0.93	775.90	
**6**	F	13.17	57.19	1.05	0.82	495.50	34.31
	R	20.05	77.30	1.06	0.92	754.03	
**14**	F	12.29	55.89	1.06	0.78	462.20	35.51
	R	19.06	74.70	1.07	0.90	716.58	
**24**	F	12.01	52.09	0.98	0.88	451.75	30.77
	R	17.35	70.70	1.02	0.91	652.20	

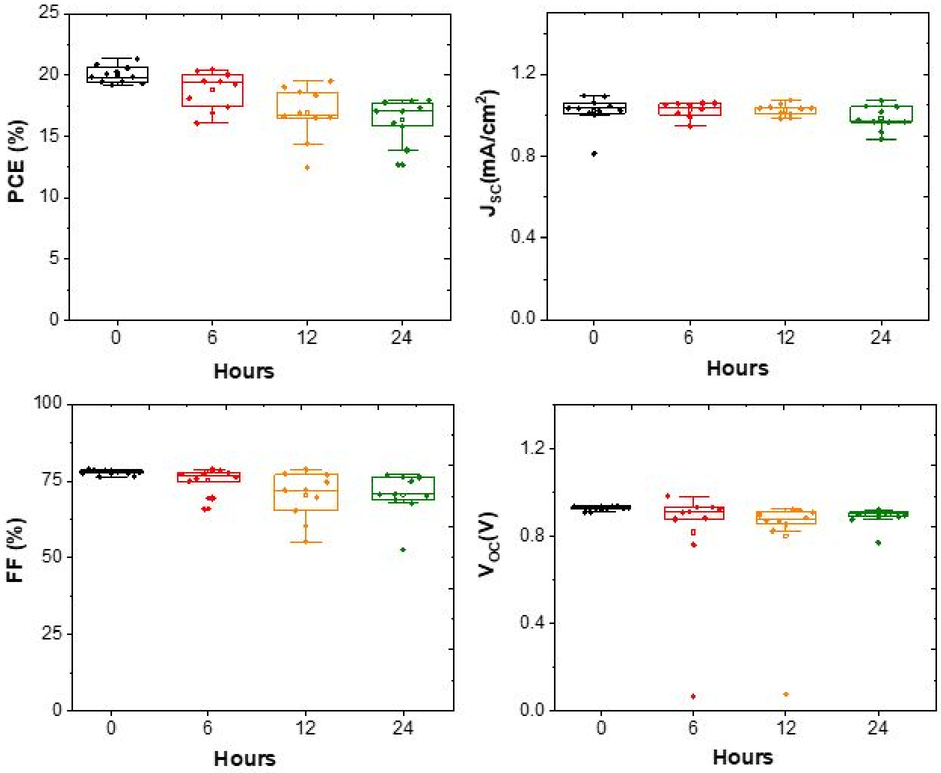

Figure S4. Statistical distribution of the solar cells [FTO/SnO_2/$MAPbI_3$/Spiro-MeOTAD/Ag] under near-UV illumination (150 lux), measured after near-UV (100 lux) pre-treatment for different time durations.

Table S3. PL decay components of near-UV (100 lux) pre-treated $CH_3NH_3PbI_3$ films on glass.

Films	τ_1 (ns)	τ_2 (ns)	A_1	A_2	τ_{avg} (ns)	Chi.sq
0 hour	1.52±0.001	118.25±0.64	1.15±2.34	1537±3.74	118.16	1164.82
6 hour	1.26±00.01	93.61±0.62	4.48±1.01	1288±4.26	93.10	870±78
14 hour	1.31±0.01	91.51±0.48	1.68±3.63	905±3.68	91.10	677.31
24 hour	0.96±0.00	92.01±1.24	3.23±8.40	353.97±2.92	28.48	463.37

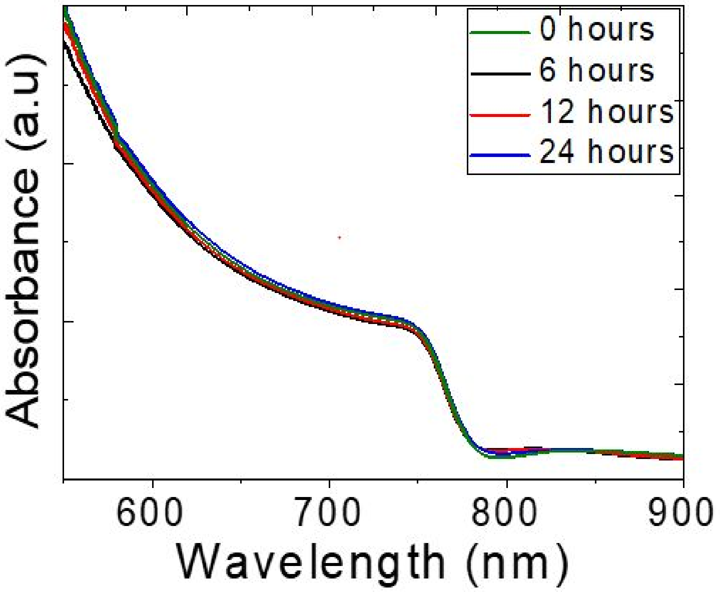

Figure S5. Optical absorption spectra of $CH_3NH_3PbI_3$ films on glass substrate measured after near-UV (100 lux) pre-treatment for different time durations.

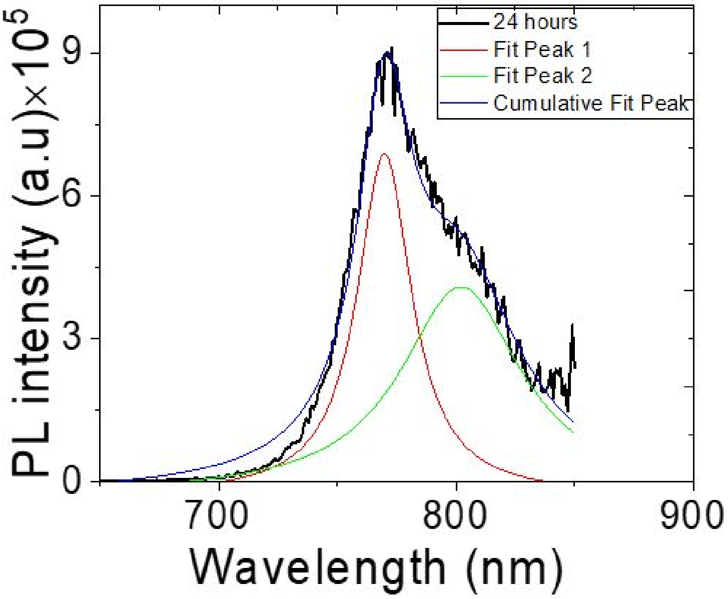

Figure S6. PL spectra (with Lorentz fitting) $MAPbI_3$ film pre-treated near-UV light (100 lux) for 24 hours.

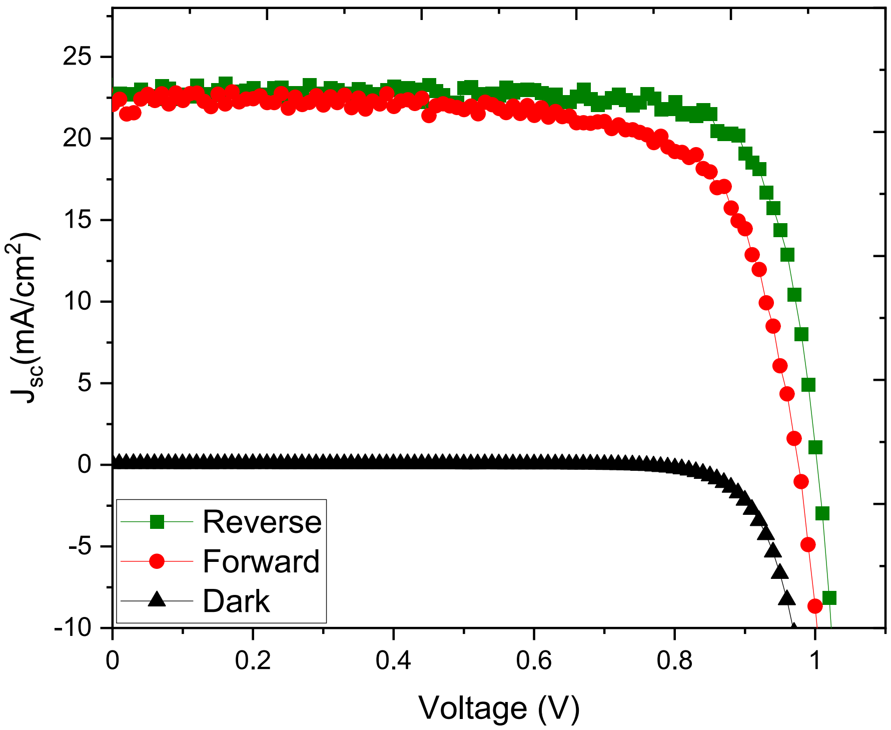

Figure S7: J-V curves (under reverse and forward scans) of the interface modified perovskite solar cells [FTO/SnO_2/ PCBM:Bphen/$MAPbI_3$/Spiro-MeOTAD/Ag] under 1 sun illumination.

Table S4: Photovoltaic parameters of the interface modified perovskite solar cells [FTO/SnO_2/ PCBM:Bphen/$MAPbI_3$/Spiro-MeOTAD/Ag] under 1 sun illumination.

Scan	PCE (%)	FF (%)	Jsc (mA/cm^2)	Voc (V)
Reverse	18.29	79.80	22.85	1.00
Forward	15.78	71.77	22.53	0.98

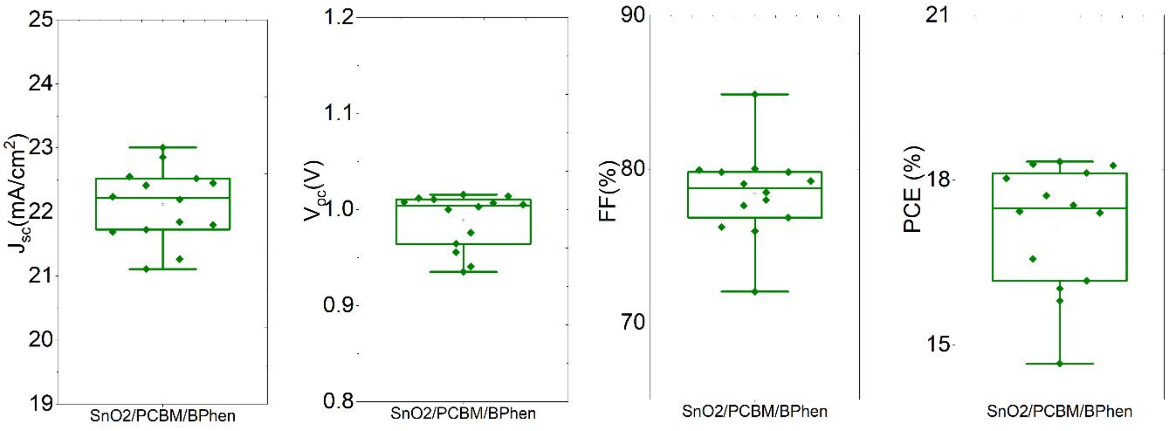

Figure S8: Statistical distribution of the perovskite solar cells with SnO_2/PCBM:Bphen electron transport layer measured under 1 sun (reverse scan).

Table S5: Series and shunt resistance of the solar cells with SnO_2 and SnO_2/PCBM:Bphen ETL (reverse scan).

**ETL**	**R_{sh} (Ω.cm^2)**	**R_S (Ω.cm^2)**
SnO_2	223.62	2.80
SnO_2/PCBM:Bphen	1359.83	2.29
